# Fabrication and *in vitro* characterization of curcumin film-forming topical spray: An integrated approach for enhanced patient comfort and efficacy

**DOI:** 10.12688/f1000research.142860.1

**Published:** 2024-02-22

**Authors:** Amitha Shetty, Akhilesh Dubey, Jeshma Chrystle, Manohar M, Anish John, Amitha N, Paramita Das, Srinivas Hebbar

**Affiliations:** 1 Department of Pharmaceutics, Nitte (Deemed to be University), NGSM Institute of Pharmaceutical Sciences, Deralakatte, Mangalore, Karnataka, 575018, India; 2 Department of Pharma chemistry, Krupanidhi College of Pharmacy, Bangaluru, Karnataka, 560035, India; 3 Department of Pharmaceutics, Manipal College of Pharmaceutical Sciences, Manipal Academy of Higher Education, Manipal, Karnataka, 576104, India

**Keywords:** film-forming spray, curcumin, design of experiment, docking study, permeation, stability study

## Abstract

**Background:**

Curcumin, known for its anti-inflammatory properties, was selected for the developing consumer friendly film forming spray that offers precise delivery of curcumin and and improves patient adherence.

**Methods:**

An optimized film-forming solution was prepared by dissolving curcumin (1%), Eudragit RLPO (5%), propylene glycol (1%), and camphor (0.5%) in ethanol: acetone (20:80) as the solvent. The solution was filled in a spray container which contained 70% solutions and 30% petroleum gas. *In-vitro* characterization was performed.

**Results:**

Potential anti-inflammatory phytoconstituents were extracted from the PubChem database and prepared as ligands, along with receptor molecules (nsp10-nsp16), for molecular docking using Autodock Vina. The docking study showed the lowest binding energy of -8.2 kcal/mol indicates better binding affinities. The optimized formulation consisted of ethanol:acetone (20:80) as the solvent, Eudragit RLPO (5%) as the polymer, propylene glycol (1%) as the plasticizer, and camphor oil (0.5%) as the penetration enhancer. The optimized formulation exhibited pH of 5.8 ± 0.01, low viscosity, low film formation time (19.54 ± 0.78 sec), high drug content (8.243 ± 0.43 mg/mL), and extended *ex vivo* drug permeation (85.08 ± 0.09%) for nine hours. Consequently, the formulation was incorporated into a container using 30% liquefied petroleum gas, delivering 0.293 ± 0.08 mL per actuation, containing 1.53 ± 0.07 mg of the drug. The film-forming spray exhibited higher cumulative drug permeation (83.94 ± 0.34%) than the marketed cream formulation and pure drug solution after 9 h, with an enhancement ratio of 14. Notably, the film-forming spray exhibited no skin irritation and remained stable for over three months.

**Conclusions:**

The developed curcumin film-forming system is promising as a carrier for wound management because of its convenient administration and transport attributes. Further *in vivo* studies are required to validate its efficacy in wound management.

## Introduction

A wound is an abrupt or gradual loss of skin or tissue integrity that causes inflammation and the entry of pathogens into the body. Wounds must heal rapidly to reduce the risk of infection and restore the functional status of the skin. In general, the healing process of a wound lasts from 8 to 12 weeks; however, for chronic wounds, it may take years. The natural wound healing process has four distinct phases: hemostasis followed by inflammatory phases. Within a few hours of injury, a new epithelium begins to develop, including the growth of new blood vessels, collagen fiber implantation, and granulation tissue production. The production of collagen is stimulated, as well as collagen reassembly and the development of scar tissue, in the final stage of wound healing. ^1^ The currently available products function in wound healing in terms of antimicrobial agents, anti-inflammatory agents, and certain drugs that promote cell proliferation to repair damaged cells.

The importance of herbal-based drug delivery systems has significantly increased in recent years. World Health Organization (WHO) estimates that more than 80% of people worldwide depend on traditional medical practices for primary healthcare. The use of phytoconstituents such as curcumin, silymarin, and quercetin has expanded over the years because of the escalating cost of drugs and the side effects of synthetic agents. ^2^ Curcumin is a polyphenolic constituent obtained from the rhizomes of *Curcuma longa* (turmeric) belonging to the family Zingiberaceae. As per the Indian Ayurvedic medicinal system, *Curcuma longa* and its constituents are widely used in several dosage forms for various disease conditions. According to other studies, polyphenol curcumin possesses potent antioxidant, antimicrobial and anti-inflammatory properties, and it is involved in re-epithelization by enhancing fibroblast proliferation. ^3^ The anti-inflammatory properties of curcumin inhibited the production of two significant cytokines, tumor necrosis alpha and interleukin -1. Upon topical administration, the antioxidant property of curcumin has been shown to drastically reduce wound healing time by scavenging free reactive oxygen molecules such as hydrogen peroxide (H202) and superoxide (O2-). ^4^ Additionally, Mohanty *et al*. demonstrated that a transdermal patch with curcumin-loaded oleic acid promoted granulation of new tissue and fibroblast infiltration within a week of application compared to the control group of animals. ^5^ Despite these benefits, curcumin has significant limitations, such as poor aqueous solubility, a higher rate of elimination, and faster degradation. Therefore, topical product formulations aim to solubilize curcumin while protecting it from hydrolysis and ensuring its prolonged release into the target location. One disadvantage of topical curcumin is that toxicity at the wound site can occur at high concentrations. Therefore, the optimal concentration of the formulation was selected. ^6^

Superficial and shallow wounds were effectively treated with polymeric films composed of transparent polyurethanes. Transparent films enable the exchange of carbon dioxide and oxygen between the wound and the environment, while protecting the site from microbial contamination. ^7^ The film can be developed by spraying drug-polymer mixtures onto the skin as a patient-friendly method compared to gels and ointments. The advantages of film-forming spray over conventional topical formulations are a uniform distribution of the drug, lower irritation, improved wound healing by controlling moisture, and continuous spraying, which enhances the mechanical properties of the polymeric film. ^8^ The nature of the polymer, nozzle type, spray pressure, and other variables can affect the spray pattern. Various film-forming sprays are available, such as ordinal spray, metered-dose spray, electrostatic spray, and ultrasonic spray.

In recent years, the discovery of new therapeutic agents has become simpler through the application of computer-assisted approaches. Determining the possible molecular interactions for selective binding between a drug and protein receptors is made easier using molecular docking techniques. These prospective approaches provide promising results at shorter intervals. ^9^ In the present study, curcumin compounds were analysed using *in silico* methods to assess their possible inhibitory activities against the nsp10-nsp16 receptor. However, *in vitro in vivo* methodologies are required to authenticate drug properties. The objective of this *in vitro* study was to develop and assess a polyphenol curcumin-based film-forming spray intended to efficiently treat wounds.

## Methods

### Materials

Curcumin was acquired from Sigma-Aldrich Chemicals Pvt. Ltd. (>94% purity) (Bangalore, India). Eudrgait S100, Eudrgait RS100, Eudrgait E100, PVP K30, Carbopol 934-P, PEG 400, propylene glycol, camphor oil, dibutyl phthalate, menthol, ethanol, and acetone were purchased from Loba Chemie Pvt. Ltd. (Mumbai, India). Transcutol P was obtained from Nice Chemical Pvt. Ltd. (Kerala, India). All other chemicals and reagents used were of analytical grades.

### Laboratory animals

The NGSM Institute of Pharmaceutical Sciences Institutional Animal Ethics Committee (IAEC) approved the purchase of adult male Wistar rats weighing approximately 200–250 g from the Nitte University-affiliated Centre for Animal Research and Experimentation (NUCARE) (Approval No. NGSM/IAEC/2021-22/240 dated 28 ^th^ June 2021). Experiments were conducted in accordance with the Committee for Control and Supervision Experiments on Animals (CPCSEA) rules, experiments were conducted. All efforts were made to ameliorate any suffering of animals by the application of methylated spirit as an antiseptic to prevent infection and careful, gentle handling to minimize distress. The nine animals allocated to three groups of three animals each were maintained in a cage, with the optimum conditions of 25 ± 2°C, 50 ± 1% relative humidity (RH), and 12-hour light/dark intervals with regular feeding, including water and pellet food (Krishna valley Aggrotech, Sangli, Maharashtra, India).

### Ligand preparation and molecular docking

Ligand preparation and molecular docking from PubChem database chemical structures of potential phytoconstituents with anti-inflammatory properties, namely both ligand and receptor molecules (nsp10–nsp16), were prepared in Autodock software (version number 4.2.0) to predict the target receptor by performing blind docking. A grid of 60 points each in the X, Y, and Z directions was chosen to accommodate any possible ligand-receptor complex using a blind docking approach. The graphical user interface of the PyMOL tool (version number 2.5) was used to visualize the structure files, and the protein-ligand interaction was prepared in Biovia Discovery Studio (2021). ^10^

### Screening of solvents

Solvents such as ethanol, isopropyl myristate, ethyl acetate, propylene glycol, oleic acid, and acetone were used to determine the solubility of curcumin in these solvents. Excess curcumin was dissolved in 100 mL of each solvent and kept in a shaking water bath for 48 h. After 48 h, the solvents were centrifuged for 15 mins at 3000 rpm. From the centrifuged sample, 1 mL was taken, diluted to 10 mL with the respective solvents, and spectrophotometrically determined at 426 nm. ^11^

### Screening of polymers

Polymers such as Eudragit RS100, Eudragit RLPO, Eudragit S100, Eudragit E100, PVP K-30, Carbopol 934-P, and PEG 400 were selected for the study. A film-forming solution was prepared using 1% curcumin and 5% polymer in ethanol: acetone (20:80), respectively, and was evaluated for viscosity, drying time, and stickiness. ^12^

### Screening of plasticizers

Plasticizers, such as PEG 400, propylene glycol, and dibutyl phthalate, were selected for this study. The polymeric solvent system was prepared by dissolving 1% curcumin and 5% Eudragit RLPO in ethanol:acetone (20:80), respectively. Subsequently, plasticizers (0.75%) were added to the previously prepared polymeric solvent system and further evaluated for flexibility. ^13^

### Screening of penetration enhancers

Penetration enhancers, such as camphor, menthol, and Transcutol P, were selected for this study. The polymeric solvent system was prepared to comprise 1% curcumin, 5% Eudragit RLPO, and 0.75% propylene glycol in ethanol:acetone (20:80), and 0.75% of each penetration enhancer, which was then evaluated for *ex vivo* permeation studies, flux, and enhancement ratio. ^14^

### Optimization by Design of Experiment

The impact of selected independent variables including concentration of polymer (A), concentration of plasticizers (B), and concentration of penetration enhancers (C), and the dependent variables included film formation time (s) and *ex vivo* permeation studies (%). The experimental conditions were optimized using Design Expert software (Version 13.0.3.0, Stat-Ease, Minneapolis, MN 55413, USA) employing central composite design (CCD). An open-access alternative that can perform an equivalent function is R commander. The levels at which the independent variables were selected are listed in Table 1. ^15^ The variable levels were optimized based on the desirability function approach. As shown in Table 2, appropriate constraint (target) levels were chosen to achieve the desired responses of the formulation.

**Table 1.** Independent and dependent factors selected and levels for the experiment.

Independent factors	Name level	Level (-1)	Level (0)	Level (+1)
A	Concentration of polymer	2.5	5	7.5
B	Concentration of plasticizer	0.5	0.75	1
C	Concentration of penetration enhancer	0.5	0.75	1
**Dependent factors**				
X1	Film formation time			
X2	*Ex vivo* permeation studies			

**Table 2.** Criteria for numerical optimization and optimized formula.

Name	Goal	Lower limit	Upper limit	Lower weight	Upper weight	Importance
A: Concentration of polymer	is in range	2.5	7.5	1	1	3
B: Concentration of plasticizer	is in range	0.5	1	1	1	3
C: Concentration of penetration enhancer	is in range	0.5	1	1	1	3
Film formation time	is target = 20	18.2	49.73	1	1	3
% cumulative drug permeation	is target = 85	65.08	89.64	1	1	3
**Optimized formulation**	**Concentration of polymer**	**Concentration of plasticizer**		**Concentration of penetration enhancer**		**Desirability**
	5%	1%		0.5%		0.98

### Formulation of optimized film-forming solution

As per the optimized formula obtained after optimization, the optimized film-forming solution was prepared by dissolving curcumin (1%), Eudragit RLPO (5%), propylene glycol (1%), and camphor (0.5%) in ethanol:acetone (20:80) as the solvent.

### Characterization of optimized film-forming solution

***Viscosity***

Viscosity assessment of the film-forming solution was assessed at a controlled temperature of 25 ± 1°C using a Brookfield viscometer (DV-11 +pro). A specific spindle (S 63) was used, and measurements were taken at different rotational speeds (10, 50, and 100 rpm) for 10 min. This systematic evaluation aimed to determine the viscosity limits suitable for the sprayability of a solution, which is an essential characteristic for its practical application. Each viscosity measurement was performed in triplicates. ^16^

***Film formation time***

The film formation time, a critical parameter for ensuring patient compliance, was assessed by placing a minute quantity of the film-forming solution onto a petri dish. The time taken for the solution to transform into a fully formed dry film was carefully observed and recorded. This evaluation aimed to determine the time required for the film-forming process, which is a crucial factor for the convenience and usability of the formulation for patients. Among the various formulations, preference was given to those that exhibited shorter drying times, as they offered quicker film development, potentially enhancing patient comfort and adherence. ^17^ Each measurement was performed thrice.

***Stickiness***

The stickiness evaluation involved gently placing a piece of cotton onto each dried film without applying any pressure. The degree of stickiness was determined based on the extent of cotton fibre retention on the film’s surface: films with a greater amount of retained cotton fibers were categorized as “very sticky,” films with a moderate level of retained fibers were classified as “medium sticky,” and films with no adherence of cotton fibers were considered “non-sticky.” This approach provides a practical and visual means of distinguishing between different levels of stickiness, systematically facilitating characterization of the adhesive properties of the films. ^18^

***pH determination***

The pH of the film-forming solution was determined using a digital pH meter (Systronics, Ahmedabad, India). The pH meter electrode was submerged in the film-forming solution, and the pH value was directly recorded from the meter’s display. ^19^

***Drug content studies***

The drug content was carried out to determine the distribution of the drug in the film-forming patch. A 2 cm patch was cut, dissolved in 5 mL methanol, and made up to a volume of 10 mL using phosphate buffer (pH 7.4) (1st dilution). From this dilution, 1 mL was pipetted into another 10 mL volumetric flask, and the volume was made using phosphate buffer (pH 7.4). Drug content was estimated spectrophotometrically using a UV-visible spectrophotometer at 426 nm. ^20^

***Ex vivo permeation study***

Drug permeation from the subcutaneous layer of the skin was assessed using porcine ear pinna skin, because the porcine ear skin membrane morphology is similar to that of human skin using Franz diffusion cells. Skin was bought from the slaughterhouse and preserved in phosphate-buffered saline (pH 7.4) at room temperature. The hair present in the skin was gently removed and dipped in hot water for 15 min to remove any subcutaneous fatty layer. The excised skin was mounted between the donor and receptor compartments of the diffusion cell apparatus with effective permeation area of 3.14 cm ^2^ and the formulation being applied on the stratum corneum facing outward of the donor compartment and the Phosphate buffer with pH 7.4 as receptor medium. A weighed amount of the formulation equivalent to 20 mg of the drug was placed on the surface of the skin. The dissolution medium was mixed using a magnetic stirrer at 50 rpm, and the temperature was maintained at 37 ± 0.5°C to simulate external skin conditions using a water bath. A sample of 1 mL was withdrawn at predetermined time intervals (0, 1, 2, 3, 4, 5, 6, 7, 8, and 9 h). The sink condition was maintained by replacing the sink with the same amount of saline buffer. One millilitre of the withdrawn sample was dissolved in 10 mL of phosphate-buffered saline (pH 7.4) in a volumetric flask. Absorbance was measured using a UV-visible spectrophotometer at 426 nm. The cumulative amount of drug that permeated (%) through the skin was plotted versus time (h). Data are expressed as the mean ± standard deviation (SD) of three determinations. This study assessed and compared the transdermal permeation capabilities of an in-house pure curcumin drug solution, film-forming spray formulation, and commercially available cream. ^21^

***Permeation data analysis***

The experimental determination of permeation parameters involved calculating the skin flux (J) in μg/cm ^2^/h using Equation (1), derived from the slope of the linear segment in the plot of cumulative amount permeated per unit area against time. The skin flux (J) can be experimentally determined using the formula

$$J=\left(\mathrm{dQ}/\mathrm{dt}\right)/A$$ (1)

where ‘J’ represents the flux (μg/cm ^2^/h), ‘A’ is the area of skin tissue (cm ^2^) through which drug permeated, and (dQ/dt) is the slope obtained from the linear curve of the plot- cumulative amount permeated per unit area versus time. Subsequently, the permeability coefficient (Kp) (cm/h) was calculated using equation (2) in which ‘C _0_’ indicate the drug concentration in the donor cell. The enhancement ratio (ER) using the following equation 3. The permeation parameters were calculated using these previously reported equations ^22^

$$\mathrm{Kp}=\frac{J\;}{\left(C0\right)}$$ (2)

$$\mathrm{ER}=\frac{\;\mathrm{Kp}\;\text{with penetration enhancer}}{\;\mathrm{Kp}\;\text{without penetration enhancer}}$$ (3)

***Verification of optimization***

The predicted results from the optimization were compared with the experimental values were compared and the relative percentage error was determined.

### Filling of film-forming solution into the container

**Figure 1.**
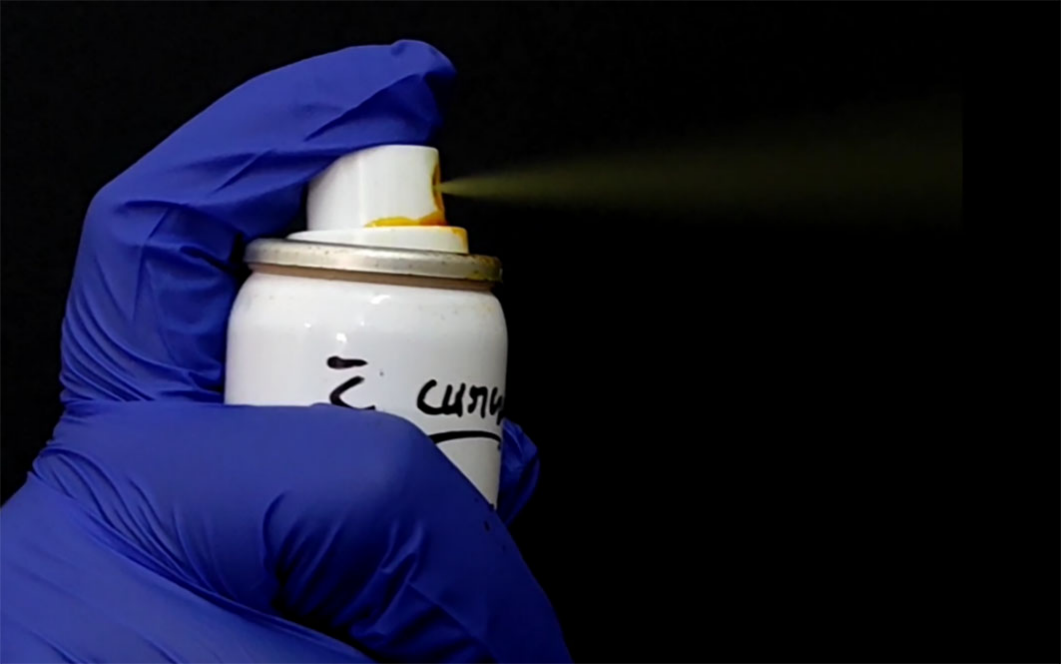
Film-forming spray of curcumin.

The process of filling the container with film-forming solutions involved the application of a pressure-filling method. This procedure employed 70% of the film-forming solution and 30% of liquefied petroleum gas as the propellant. This precise mixture was introduced into the container to ensure controlled and efficient filling. The developed formulation is illustrated in Figure 1.

### Evaluation parameters related to the container

***The delivery volume per actuation***

The formulation delivered per actuation was measured using the following formula:

$$\mathrm{AL}\frac{\left(\mathrm{Wo}–\mathrm{Wt}\right)}{\mathrm{Dn}}$$ (4)

Where AL represents the volume of the solution delivered per actuation, Wo and Wt is the weight of the formulation before and after actuation, and Dn is the density of the formulation. The measurements were made in triplicates and the results were expressed as mean ± SD. ^23^

***Spray pattern***

The formulation was sprayed onto Whattmann paper positioned at a predetermined distance. This paper was securely fastened to a board to create a stable platform for the experiment. The spray formulations were discharged from the container at a distance of 15 cm from the paper surface. This standardized procedure allowed for the observation and assessment of the resultant spray pattern on paper. By conducting this test, the uniformity and spread of the spray could be visually examined and analysed for consistency and effectiveness. ^24^

***Spray angle***

The sprays were actuated horizontally on white paper mounted 15 cm from the nozzle. The radius of the circle formed on the paper was recorded in triplicate from different directions. The spray angle (θ) is calculated using the following equation:

$$\text{Spray angle}\;\left(\theta \right)=\tan^{-1}\;\left(l/r\right)$$ (5)

where l is the distance of the paper from the nozzle and r is the average radius of the circle. ^25^

### Skin irritation studies

The protocol for the present study (Ref: NGSM/IAEC/2021-22/240) was approved by the Institutional Animal Ethics Committee (IAEC). A skin irritation study was conducted in male Wistar rats to ensure product safety. Inclusion and exclusion criteria for animals included factors such as the health status, age, weight, of either sex were considered. Nine healthy male Wistar rats weighing between 200–250 gm were included based on a thorough literature survey, aligning with ethical guidelines to use the minimum animals necessary while ensuring statistical robustness. The sample size was determined according to the available literature, as the study did not consider a specific primary outcome measure for hypothesis testing, and therefore no a priori sample size calculation was conducted. Nine Wistar rats were divided into three groups, with three rats in each group: Group I (control, placebo spray), Group II (standard, drug in solvent), and Group III (film-forming formulation). They were caged separately according to their groups and provided with food, water, and proper environmental conditions. Twenty-four hours before the application, the hair on the dorsal side (3 × 3 cm) of the male Wistar rats was removed using an electric clipper in the direction of the tail to the head without damaging the skin, and the methylated spirit was then applied as an antiseptic to the shaved region with the aid of cotton wool to prevent infection caused by bacteria. As only skin irritation studies were carried out on the dorsal surface, animals were not euthanised. The formulations were applied to the depilated areas of the animals in each group. Exclusion criteria included any signs of pre-existing skin conditions or illness. For data points, criteria for inclusion or exclusion involved the presence of observable skin irritation reactions. Humane endpoints were based on the observations such as presence of severe irritation, ulceration, and significant behavioural changes. If these conditions transpired, the animals were excluded from the study with immediate implementation to minimize suffering. Skin irritation was observed according to the OECD guidelines at predetermined intervals three times daily. At the end of 24 h, the skin surface was rinsed with distilled water and graded if edema was observed. Wistar rats were re-caged, and post-procedure observation (i.e., scoring) was repeated for seven days. Based on the scoring, the formulated films were graded as non-irritant, irritant, or highly irritant. ^26^^,^^27^

### Stability studies of formulation

The formulated film-forming spray was kept for stability investigation according to International Council for Harmonisation (ICH) guidelines [Q1A, R2] for six months at 40°C ± 2 and relative humidity of 75 ± 2%. The study conditions were maintained in a stability chamber (Photostability Chamber, Rotek). At three months, samples were taken to determine the film-formation time, pH, volume delivered upon each actuation, and drug content per actuation. The samples were assessed in triplicate [n = 3]. ^28^

### Statistical analysis

The results are presented as mean ± SD. The experiments were performed in triplicate. A t-test and one-way ANOVA were used for statistical analysis to determine significant differences between the means tested (p <0.05), applying Sidak’s multiple comparisons test to assess the differences between groups. All calculations were completed using Graphpad Prism 10 (GraphPad Inc., La Jolla, CA, USA). An open-access alternative that can perform an equivalent function is JASP.

## Results

### Docking analysis

**Figure 2.**
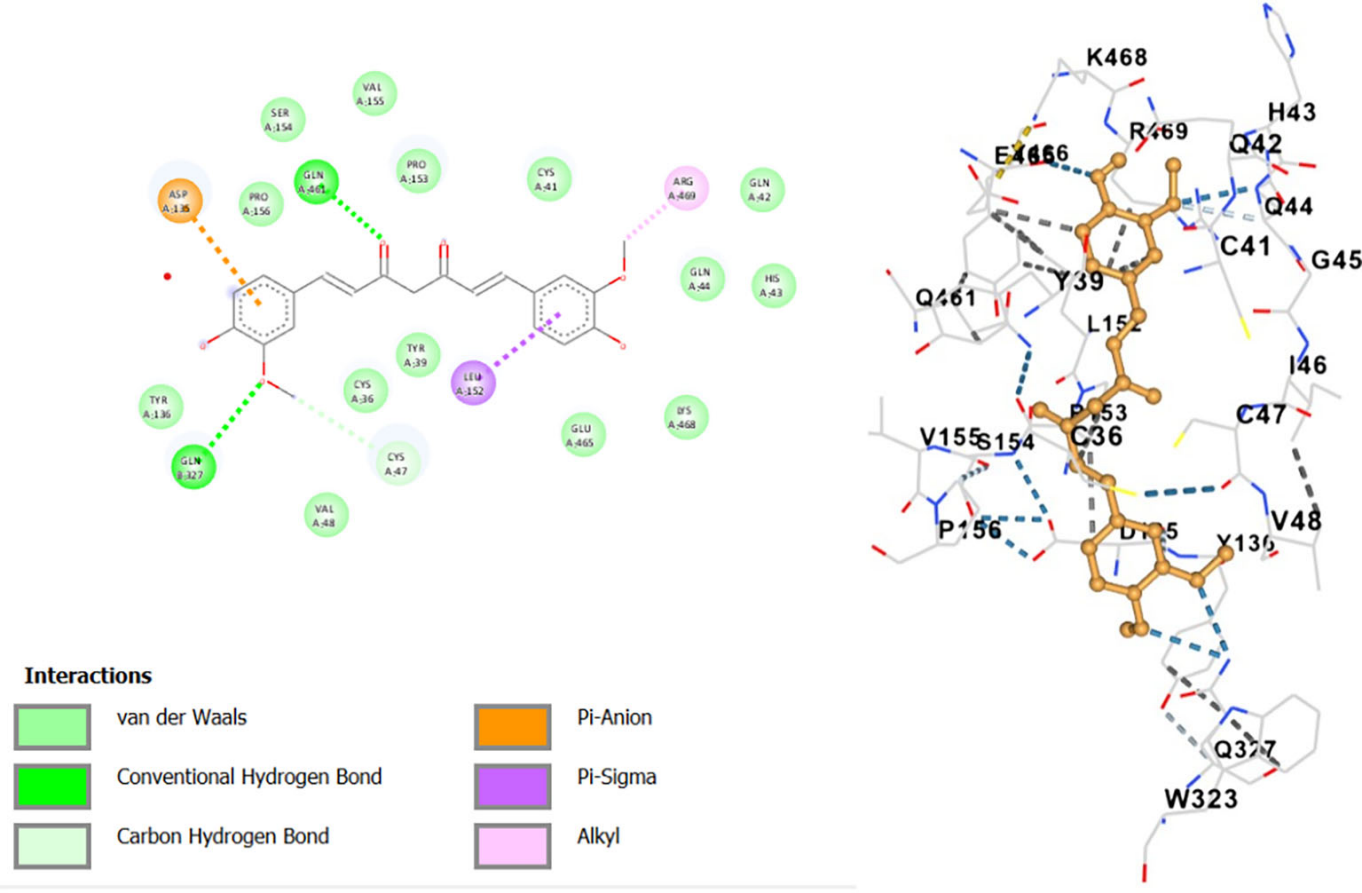
Molecular docking analysis of curcumin.

In this study, *in-silico* docking tools were employed to validate the viability of selected curcumin molecules with respect to their interactions with the target protein (nsp10-nsp16), considering a variety of conformations and binding energies. A comprehensive understanding of binding dynamics was obtained by scrutinizing multiple conformations, shown in Figure 2. This multifaceted approach revealed a spectrum of binding energies, providing insights into the strength of the interactions between the curcumin molecules and the receptor. Curcuminoids in turmeric exhibited the most favorable binding energy among the studied molecules, with a remarkable value of -8.2 kcal/mol. This outcome underscores the potential of curcumin as a potent ligand for target proteins. Through its interactions with the receptor, curcumin establishes hydrogen bonds with key residues, including ASP:135, GLN:327, CYS:47, LEU:152, and ARG:469, contributing to its robust binding affinity. A crucial indicator of the efficacy of the ligand-receptor interaction is the calculated change in Gibbs free energy (ΔG). In the context of this study, the lower value of ΔG associated with curcumin underscores its enhanced binding affinity to the target protein. This implies a more stable and robust interaction, reinforcing the potential therapeutic relevance of curcumin. ^29^ Thus, the computational docking study offers valuable insights into the molecular-level interactions between curcumin and the nsp10-nsp16 receptor.

### Screening of solvents

**Figure 3.**
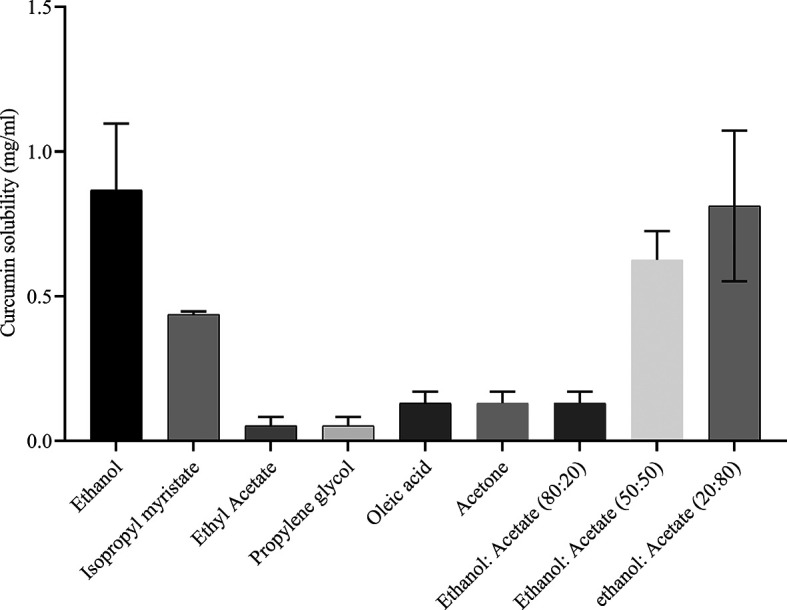
Solubility assessment of curcumin in various oils.

The solvent used to suspend a drug has the potential to exert a significant impact on the thermodynamic activity of the drug itself. This can lead to increased drug penetration into the subcutaneous layer, subsequently facilitating the achievement of a more substantial concentration of the drug at the topical application site. ^30^ Hence, various solvents, including ethanol, isopropyl alcohol, ethyl acetate, propylene glycol, oleic acid, and acetone, were systematically assessed for compatibility with curcumin. Notably, among the solvents tested, ethanol and acetone demonstrated the highest solubility for curcumin. Owing to the lone pair of oxygen atom electrons in its molecular structure, curcumin forms hydrogen bonds that allow it to dissolve in organic solvents. ^31^ Therefore, different ethanol/acetone ratios (20:80, 50:50, and 80:20) were used to determine the concentration. The solvent with the highest concentration was ethanol: acetone, taken in a ratio of 20:80, and thus was selected as the best solvent for further studies ( Figure 3).

### Screening of polymers

In the current study, the screening process for polymers revolved around critical attributes, including viscosity, drying time, and stickiness, as listed in Table 3. Often regarded as a crucial determinant, viscosity leans towards lower values for spray-based film formation, enabling accurate and adaptable dosing strategies. In this context, the formulations were thoroughly evaluated to assess viscosity, yielding a spectrum ranging from 0.21 to 2.9 cps. Consequently, solutions with lower viscosities were developed for further consideration. Drying time, another parameter, underpins the efficacy of film-forming solutions. Swift drying times, typically within the range 3–4 minutes, are preferred to prevent extended waiting periods for patients. To ascertain this, the formulations were subjected to drying time evaluations spanning from 10 s to 10 min. Subsequent analysis led to the selection of solutions with the shortest drying times, effectively avoiding prolonged patient waiting intervals. ^32^ Moreover, an imperative criterion involves the adherence potential of the prepared film-forming solutions, which necessitates minimal stickiness to avert undesired interactions with clothing or surfaces. The assessment of stickiness rendered a diverse spectrum, encompassing solutions ranging from least sticky to markedly adhesive solutions. Consequently, solutions characterized by reduced stickiness were identified as successful candidates, warranting further investigation. ^33^ An intriguing observation was made within the conducted investigations regarding Carbopol 934-P, which displayed an exceptionally rapid drying time of 10 ± 0.31s owing to its inherent hydrophilic nature. However, this was accompanied by a moderate level of stickiness. In contrast, Eudragit RLPO exhibited a marginally extended drying time of 11 ± 0.03s while remaining non-sticky, which rendered it particularly favorable. As a result, Eudragit RLPO emerged as the preferred polymer for film-forming spray formulation.

**Table 3.** Screening of polymers based on viscosity, drying time, and stickiness.

Polymers (5%)	RPM			Drying time (Seconds)	Stickiness
	Viscosity				
Eudragit RLPO	10	50	100	11 ± 0.03	+
	0.4 ± 0.01	0.37 ± 0.16	0.21 ± 0.27		
Eudragit E100	10	50	100	45 ± 0.01	+
	0.76 ± 0.37	0.54 ± 0.12	0.53 ± 0.09		
Eudragit RS100	10	50	100	50 ± 0.71	+
	0.67 ± 0.32	0.55 ± 0.19	0.31 ± 0.08		
Eudragit S100	10	50	100	28.75 ± 0.12	+
	0.74 ± 0.41	0.65 ± 0.17	0.27 ± 0.05		
PVP K- 30	10	50	100	60 ± 0.41	+++
	2.9 ± 0.26	2.4 ± 0.38	1.90 ± 0.49		
Carbopol 934-P	10	50	100	10 ± 0.31	++
	0.78 ± 0.29	0.33 ± 0.02	0.23 ± 0.07		
PEG 400	10	50	100	300 ± 0.03	+
	0.9 ± 0.27	0.6 ± 0.11	0.4 ± 0.38		

Results are presented as mean ± standard deviation (n = 3). + Sign indicates not sticky; ++ indicates medium sticky; and +++ sign indicates very sticky.

### Screening of plasticizers

Plasticizers contribute to improved film-forming solution flexibility, and thus reduce the brittleness, cracking, and flaking of the film. The plasticizers selected were PEG 400, propylene glycol, and dibutyl phthalate. Dibutyl phthalate was not considered because it was not sufficiently flexible, was very sticky, and did not form an even patch. PEG 400 and propylene glycol (0.75%) were evaluated based on flexibilities. The propylene glycol films were found to be more flexible than PEG 400.

### Screening of penetration enhancers

**Figure 4.**
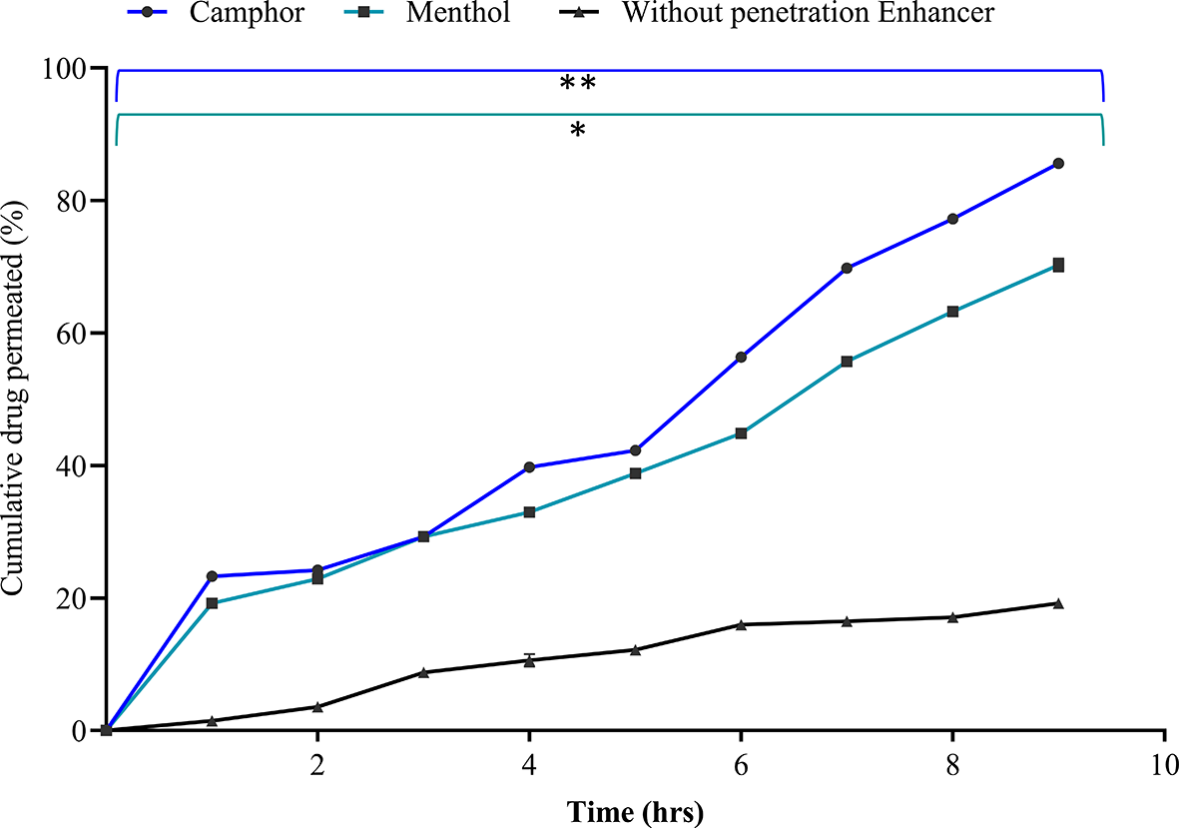
Cumulative drug release from various penetration enhancers. The data presented are the mean ± standard deviation (n = 3). Significance was measured by One-way ANOVA. ** indicates the significance between camphor and without penetration enhancer (p < 0.005), and *indicates the significance between camphor and menthol (p < 0.05).

The complex arrangement of the intercellular lipid layers is a major barrier to transdermal drug penetration. The drug diffusion process begins, and the interaction between the permeation enhancer and skin lipids influences its solubility. ^34^ The stratum corneum, sweat glands, or hair follicles are all involved in the process used by penetration enhancers to allow drugs to get through. The penetration enhancers selected were camphor, menthol, and Transcutol P at a concentration of 0.75% each. They were evaluated based on the *ex vivo* drug permeation, flux, permeability coefficient, and enhancement ratio. The addition of Transcutol P to the solution resulted in turbidity and increased viscosity; thus, it was not considered for further studies. Camphor 0.75% demonstrated statistically significant maximum drug permeation of 85.60 ± 0.32% after nine hours, with a flux value of 7.41 ± 0.17 μg/cm ^2^/hr and a permeability coefficient of 0.37 ± 0.05 cm ^2^/hr, compared to menthol (p < 0.05) with an enhancement ratio of 18.5 as depicted in Figure 4 and Table 4. Therefore, camphor was selected as the best penetration enhancer in this study.

**Table 4.** Screening of penetration enhancers based on permeation data analysis.

Parameters	Without penetration enhancer	Camphor	Menthol
Flux (μg/cm ^2^/hr)	0.47 ± 0.13	7.41 ± 0.17	6.11 ± 0.14
Permeability coefficient (cm ^2^/hr)	0.02 ± 0.01	0.37 ± 0.05	0.30 ± 0.12
Enhancement ratio	-	18.5	15.0

Results are presented as the mean ± standard deviation (n = 3).

### Optimization by Design of Experiment

The formulation was optimized using 2 ^3^ levels of factorial randomized central composite design using the Design Expert software. Seventeen trial batches were prepared and evaluated for film formation time and *ex vivo* drug permeation. The film formation time and *ex vivo* drug permeation studies were 18.20–49.73 secs and 65.08–89.64%, respectively ( Table 5).

**Table 5.** Skin irritation study.

Groups	Type	Parameter		Primary irritation index
		Erythema	Edema	
Group I	Control group	-	-	-
Group II	Standard group	-	-	+
Group III	Film-forming spray	-	-	-

Results are presented as mean ± standard deviation (n = 3). - Sign indicates no reaction, + indicates some reaction, +++ sign indicates more reactions.

***Impact of selected variables on film formation time***

**Figure 5.**
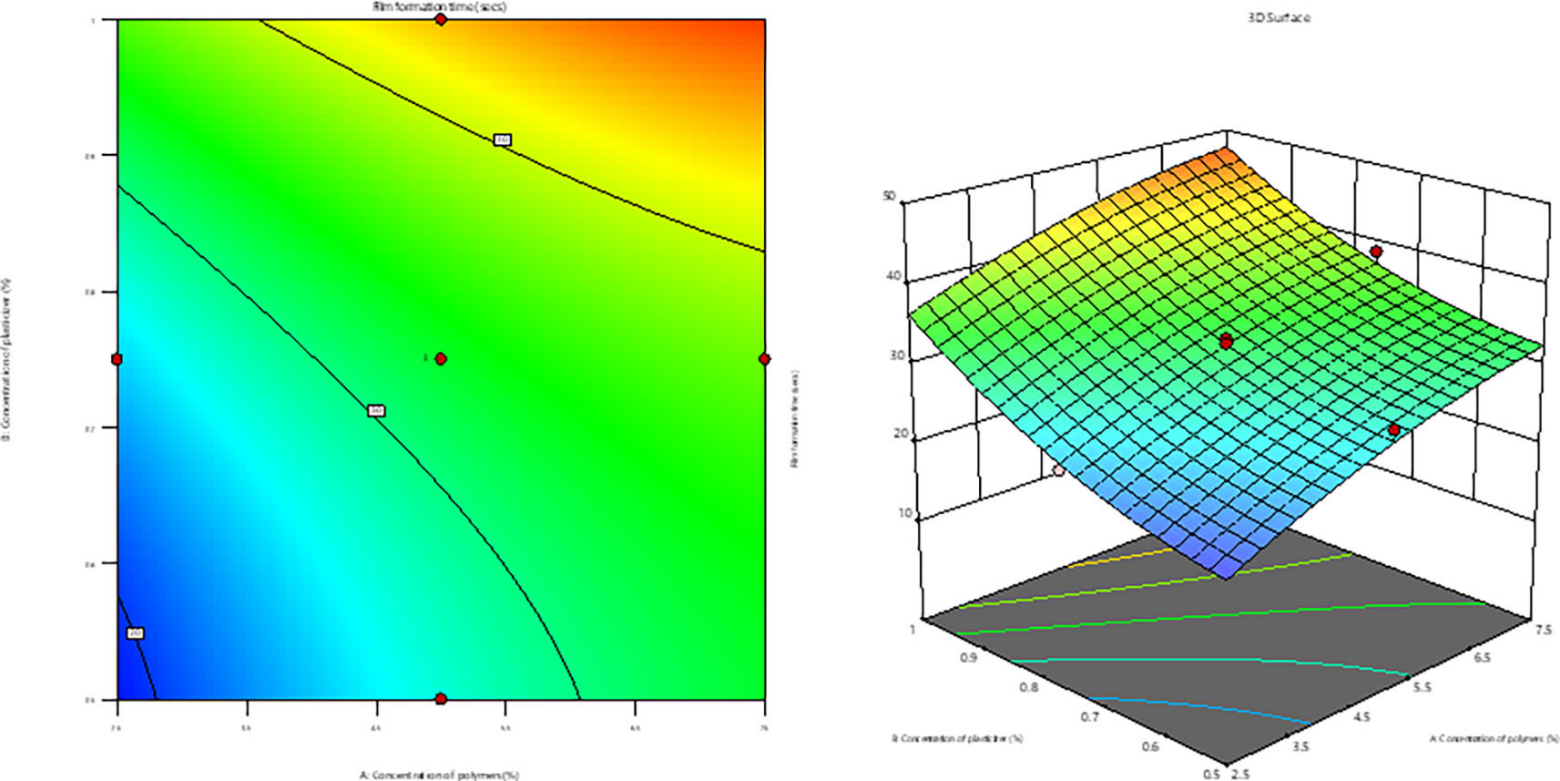
Contour plot and response surface plot of film formation time.

The p-value <0.0001 and an F-value of 69.71 indicated that the quadratic model for film formation time was significant. The non-significant lack of fit, (p = 0.5471), suggested the appropriateness of the model for predicting film formation time. the contour plot and response surface plot ( Figure 5), indicated that the film formation time was substantially increased with increase in the concentrations of polymer, plasticizer, and permeation enhancer from -1 to +1 levels.

The impact of selected factors on the film formation time is shown in the following equation:

$$\text{Film formation time}=+32.67+6.29A+8.20B+1.61C-0.6063\mathrm{AB}+0.3712\mathrm{AC}+0.3412\mathrm{BC}-1.67A^{2}+2.87B^{2}+0.0494C^{2}$$ (6)

A, B, and C represent the coded values for the polymer, plasticizer, and penetration enhancer concentrations, respectively.

***Impact of selected variables on ex vivo drug permeation studies***

**Figure 6.**
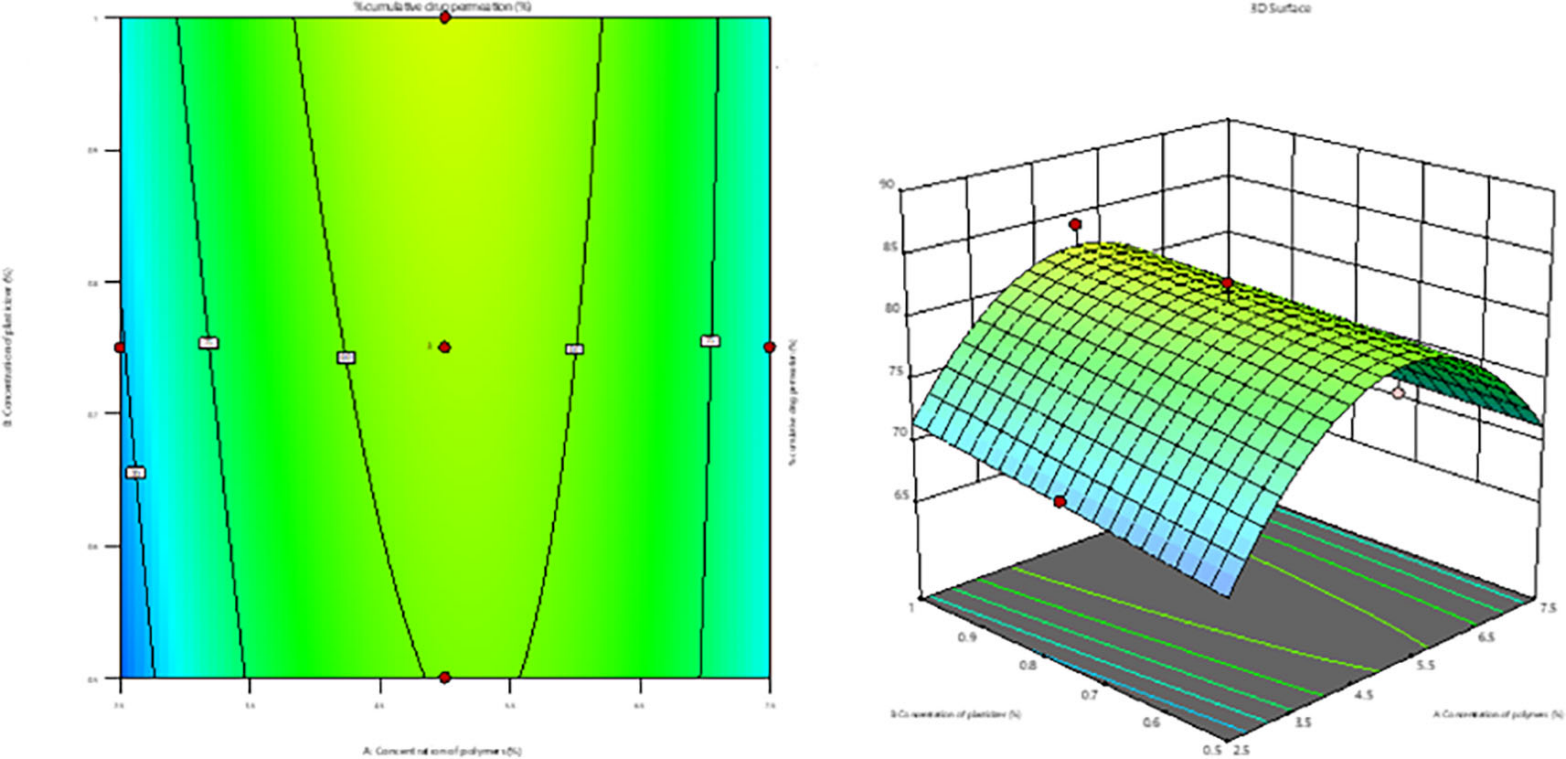
Contour plot and response surface plot of *ex vivo* drug permeation.

The p-value of 0.0036 and an F-value of 9.53 indicated that the quadratic model for *ex-vivo* drug permeation studies was significant. The non-significant lack of fit, ( *p*=0.1455), indicated that the appropriateness of the model for *ex-vivo* drug permeation studies. The contour plot and response surface plot ( Figure 6) indicated significant increase in *ex-vivo* drug permeation with an increase in both polymer and plasticizer concentrations from -1 to +1 level. However, as the penetration enhancer increased from 0.5% to 1%, the *ex vivo* drug permeation studies decreased. These factors significantly affected *ex vivo* drug permeation studies, as shown in the following equation:

$$\text{Film formation time}=+81.34+0.9780A+1.13B-3.50C-0.8175\mathrm{AB}+1.33\mathrm{AC}-0.1700\mathrm{BC}-10.69A^{2}-0.0613B^{2}+1.17C^{2}$$ (7)

A, B, and C represent the coded values for the polymer, plasticizer, and penetration enhancer concentrations, respectively.

The criteria for numerical optimization and the optimized formulation based on Design Expert software are presented in Table 2.

### Characterization of optimized film-forming solution

The pH, viscosity, film formation time, drug content, and *ex-vivo* permeation studies were performed as described in the methods section. The results are shown in Table 6. The colour and appearance of the optimized film-forming solutions were then evaluated. The physical appearance of the film-forming solution was dark yellow and transparent. The evaluation of pH is a crucial parameter for topical formulations, as it can cause skin irritation if it differs from standard skin pH conditions (i.e., 4 to 6). The pH was 5.8 ± 0.01, closer to the skin pH. Therefore, the curcumin film-forming solution may not irritate the skin while administered topically and also helps in the ionization of the drug into the permeation process. The viscosity of the optimized film-forming solution was 2.268 cps, which ensured accurate and flexible dosing because viscosity is inversely proportional to the number of polymers utilized; a higher viscosity results in less spray coverage.

**Table 6.** pH, viscosity, film formation time, and drug content of the optimized formulation.

pH determination	Viscosity measurement (cps)	Film formation time (Seconds)	Drug content (mg/ml)	Cumulative drug permeation after 9 h (%)
5.8 ± 0.01	2.268 ± 0.57	19.54 ± 0.78	8.243 ± 0.43	85.08 ± 0.09

Results are presented as the mean ± standard deviation (n = 3).

The mechanism of film formation is the evaporation of the solvent base. Solvent drying at room temperature is essential to obtain a film-forming solution that promotes patient compliance. The test revealed the time required for solvent evaporation and production of skin-contacting films. The total drying time of the optimized film-forming solution was 19.54 ± 0.78 s, indicating an ideal value compared to the standard film-forming spray, that is, less than 3 min. ^35^

The drug content in the optimized film-forming solution was 8.243 ± 0.43 mg\mL. Drug content assessment suggested that the drug was uniformly distributed. Uniformity of the drug content is necessary to ensure the homogeneity of the dispersed drug in the entire formulation.

The cumulative drug permeation of the optimized film-forming solution ranged from 21.01 ± 0.06% after 1 hr to 85.08 ± 0.09% after 9 h. Hence, the optimized film-forming curcumin solution containing 5% RLPO, 1% propylene glycol, and 0.5% v/v camphor showed prolonged drug permeation.

### Comparison of experimental results with predicted responses of optimized formulation

Table 7 lists the predicted and experimental values of all response variables and the percentage error. The mean percentage error was 1.04 after comparing the observed and predicted responses. Thus, a low magnitude of error indicated an excellent fit of the model.

**Table 7.** Selected solution and % error between the predicted and the observed values.

Factors			Responses	
A	B	C	Film formation time	*Ex vivo* drug permeation
			Predicted	
5%	1%	0.5%	20.50 secs	83.95%
			Observed	
			19.54 secs	85.08%
Relative % error			0.96	1.13

### Evaluation parameters related to the container

***The volume delivered upon each actuation***

In this study, assessing the volume delivered per actuation emerged as a pivotal consideration for ensuring the reliability and uniformity of dosing across applications. The experimental findings unveiled a delivered volume of 0.293 ± 0.08 mL for each spray actuation.

***Spray pattern***

**Figure 7.**
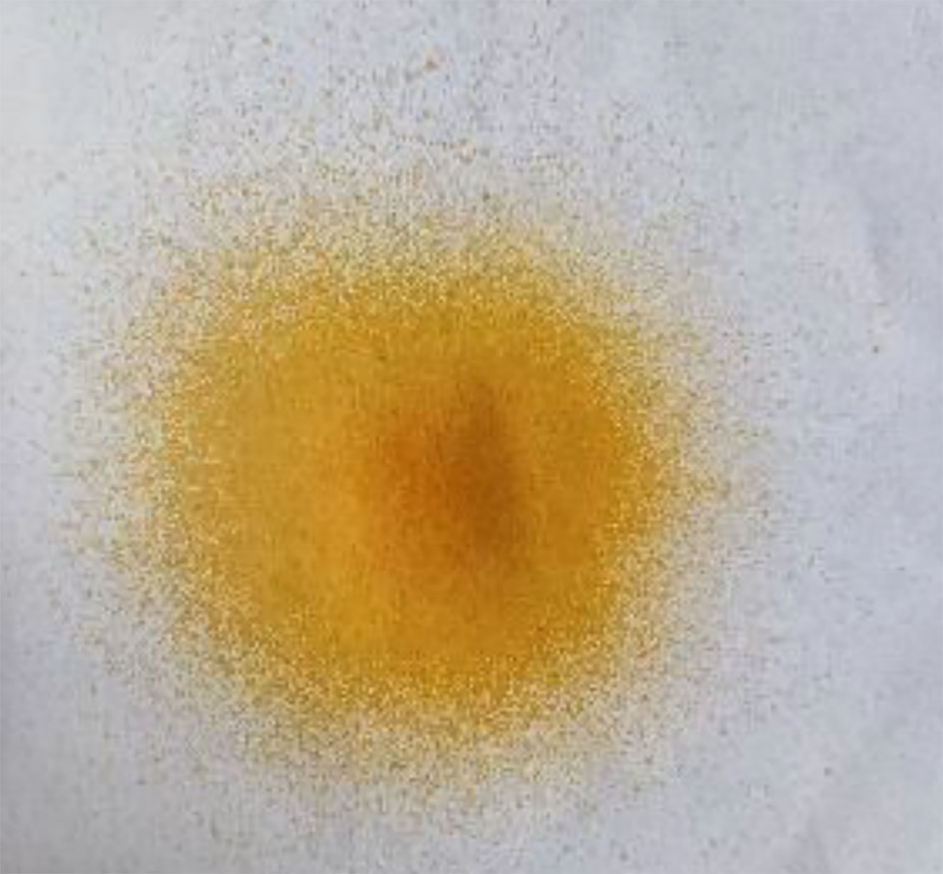
Spray pattern of the film-forming solution from the container.

Evaluation of the spray pattern is an integral aspect in assessing the functionality and performance of the optimized film-forming solution. A visual representation of the spray pattern in Figure 7 empirically confirms the performance of the formulation. This illustration provides tangible insight into the dispersion dynamics of the solution, reaffirming its potential for successful application.

***Spray angle***

The spray angle was found to be 72.59° ± 1.43, which was optimal. The actuation amount and spray angle are influenced by the nature of the polymer and its concentration, as well as the viscosity of the formulation.

***Drug content***

Determining the amount of curcumin delivered per actuation is a pivotal parameter for assessing the reliability and consistency of drug delivery systems. The findings revealed a consistent delivery of curcumin, with 1.53 ± 0.07 mg delivered per actuation.

### *Ex vivo* permeation study

**Figure 8.**
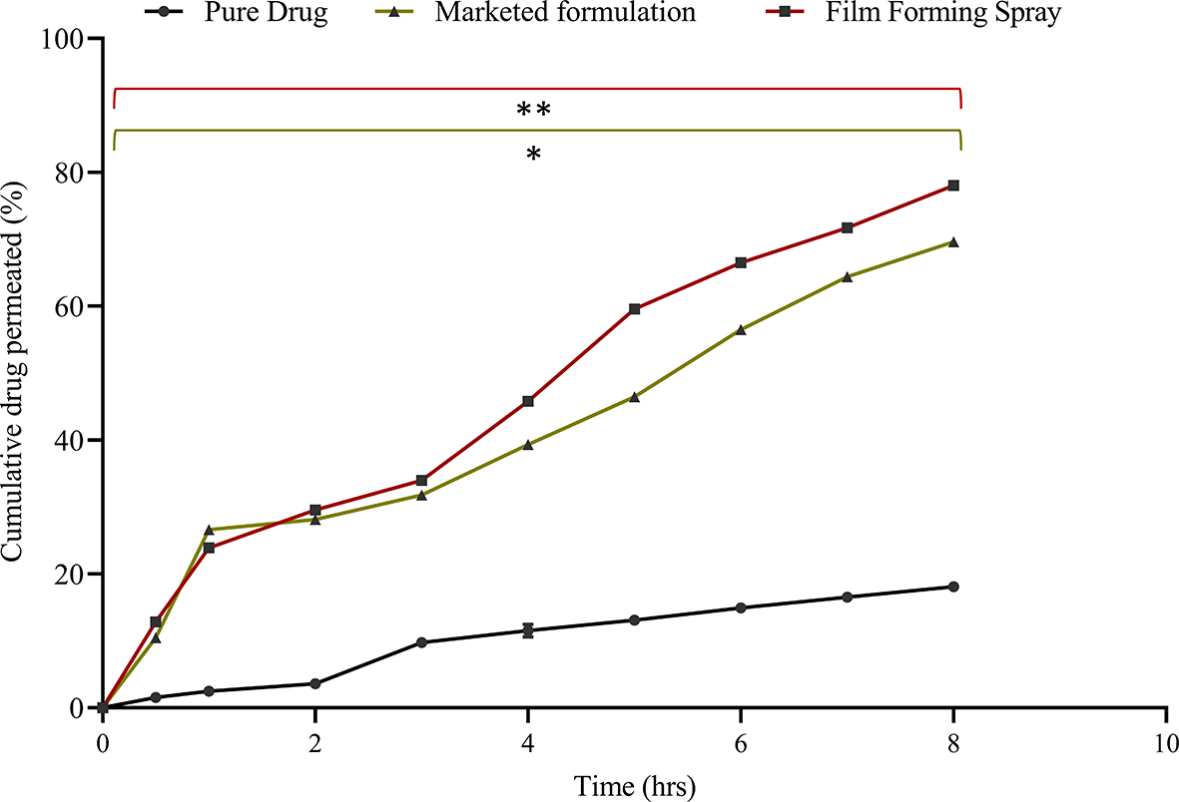
*Ex vivo* permeation study. The data represent the mean ± SD. n = 3. The data presented are the mean ± standard deviation (n = 3). Significance was measured by One-way ANOVA. ** indicate the significance between film-forming spray and pure drug (p < 0.005), and * indicate the significance between film-forming spray and marketed formulation (p < 0.05).

An *ex vivo* animal skin model is the most realistic method for evaluating transdermal formulations and understanding drug permeation through the subcutaneous skin layer. Porcine ear skin shows the most relevant histological structure as human skin, with a subcutaneous thickness of 21–26 μm and hair follicle density of 20/cm ^2^. ^36^ The Figure 8 results indicated that the film-forming spray exhibited the highest drug permeation, showing a significant difference compared to the pure drug solution (p < 0.005), with an exceptional 83.94 ± 0.34% permeation after 9 h. This was accompanied by a flux value of 8.47 ± 0.13 μg/cm ^2^/hr and a permeability coefficient of 0.42 ± 0.35 cm ^2^/hr. In contrast, owing to its physicochemical properties, the pure drug solution displayed the lowest permeability at 20.23±0.16%. The formulation of curcumin as a film-forming spray significantly elevated drug permeation compared to that of the pure drug solution, demonstrating an enhancement ratio of 14 ( Table 8). This enhancement can be attributed to a natural permeation enhancer, 0.5% camphor, which interacts with subcutaneous intracellular lipids, augments the partition coefficient of curcumin. Notably, the permeability of the film-forming spray was slightly superior (enhancement of 1.4) to that of the marketed cream formulation.

**Table 8.** Comparison of permeation data analysis of the pure drug, film-forming spray, and marketed formulation.

Parameters	Pure drug solution	Film forming spray	Marketed cream formulation
Flux (μg/cm ^2^/hr)	0.78 ± 0.09	8.47 ± 0.13	7.61 ± 0.14
Permeability coefficient (cm ^2^/hr)	0.03 ± 0.19	0.42 ± 0.35	0.38 ± 0.05
Enhancement ratio	-	14.0	12.6

Results are presented as the mean ± standard deviation (n = 3).

### Skin irritation studies

**Figure 9.**
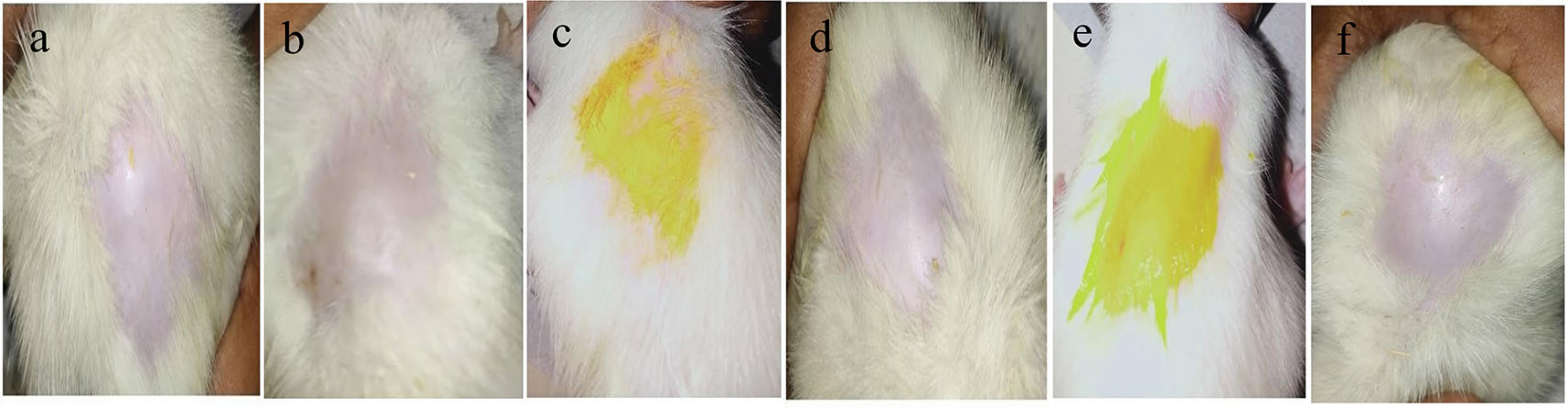
Skin irritation study (a) Control 1st day and (b) 7th day, (c) Standard 1st day and (d) 7th day, (e) Film-forming spray 1st day and (f) 7th day.

Skin irritation is a sign that the skin is exposed to various external stimuli that cause inflammation, without triggering the production of specific antibodies. ^37^ In the transdermal formulation, skin irritation may be caused by the chemical nature of the API and other excipients. The concentration of the permeability enhancers and the formulation’s contact time duration may be contributory variables. This study was conducted using healthy Wistar rats. Group I was used as the control, Group II was the standard drug group, and Group III was used for the film-forming spray. After visual inspection, the film-forming spray was compatible with rat skin and showed no irritation. The rats were observed after 0 h, 1 d, and 7 d, and readings were recorded. There were no signs of erythema or edema over the entire period of seven days. From the results tabulated in Table 9 and Figure 9, it can be concluded that the curcumin film-forming spray is irritation-free.

**Table 9.** Skin irritation study.

Groups	Type	Parameter		Primary irritation index
		Erythema	Edema	
Group I	Control group	-	-	-
Group II	Standard Group	-	-	+
Group III	Film-forming spray	-	-	-

Results are presented as the mean ± standard deviation (n = 3). - Sign indicates no reaction, + indicates some reaction, +++ sign indicates more reactions.

### Stability study

The formulation was assessed according to the ICH regulatory authority [Q1, R ^2^] accelerated stability conditions and observed changes in the formulation for its film formation capacity in terms of pH, volume delivered, and drug content per actuation using a UV spectrometer. No statistically significant difference (p ≤ 0.05) in the intermediate and the final testing point of the study (three and six months) was observed in the film formation time, pH, actuation volume, and drug content per actuation values compared to the initial formulation.

Furthermore, drug content appears to decrease non-significantly from 1.53 ± 0.07 mg/actuation to 1.50 ± 0.27 mg/actuation to 1.49 ± 0.57 mg/actuation to 1.43 ± 0.01 mg/actuation. According to the ICH Q1 R2 guidelines, these minimal changes are acceptable to pass the stability study. The formulated product maintained its quality with respect to its physical and chemical stability over six months at 40 ± 2°C and 75 ± 2% RH. The results of this study are presented in Table 10. ^38^

**Table 10.** Stability evaluation of Film-forming spray formulation.

Test parameters	Storage conditions	Relative humidity	Months			
			0 [Before stability study]	1	3	6
Film-formation time (secs)	40 ± 2°C	75 ± 2%	19.54 ± 0.78	19.41 ± 0.57	20.70 ± 0.22	20.40 ± 0.99
pH			5.8 ± 0.01	5.8 ± 0.41	5.8 ± 0.74	5.8 ± 0.53
Volume delivered upon each actuation (mL)			0.293 ± 0.08	0.190 ± 0.24	0.192 ± 0.27	0.192 ± 0.50
Drug content (mg per actuation)			1.53 ± 0.07	1.50 ± 0.27	1.49 ± 0.57	1.43 ± 0.01

Results are presented as the mean ± standard deviation (n = 3).

## Discussion

Computational docking techniques hold immense promise for predicting the potential applicability of a drug by elucidating its interactions with a specific receptor. ^39^^,^^40^ The outcomes of the docking investigations shed light on the distinctive properties of different curcumin molecules in their interactions with the target protein. Identifying the optimal binding energy of curcumin and its specific interactions with key residues furthers our understanding of its ligand potential. These findings provide a theoretical foundation for the efficacy of curcumin and pave the way for future experimental validation and potential applications in drug development or therapeutic interventions targeting the nsp10-nsp16 protein complex.

The exploration of solvent solubility is inherently tied to supersaturation, a principle pivotal to solvent evaporation from the surface of the skin post-application. The fact that drugs remain soluble in the non-volatile component prevents drug precipitation. The combined mixture of acetone and ethanol increased the vapor pressure compared with the use of a single solvent. ^41^

The selection of appropriate polymers is a pivotal factor that influences the successful formation of film-forming sprays. ^42^ The reasonable drying time and non-sticky behavior positioned Eudragit RLPO as the optimal choice for further development and evaluation within the context of this study.

A flexible wound film can bend, stretch, and adapt to body movements and changes in the wound area. This is crucial for patient comfort and effective wound healing. When plasticizer molecules are coupled with long polymeric chains, the film functionality is altered. This combination decreases the intermolecular forces and increases the flexibility of the films. ^43^ The propylene glycol structure has less polarity than PEG 400; therefore, it has a lower ability to bind water. The lower hydrophilicity of propylene glycol leads to greater flexibility of the film. ^44^

Camphor is a promising natural penetration enhancer because according to a study by Feng Xie *et al*., it exhibits a safer and more effective increase in penetration, improving the transdermal permeation of medications with various lipophilicities. ^45^

The accurate and consistent delivery of a specific volume with each actuation of a spray is a critical parameter that directly influences dosing precision within the application context. The close approximation in volume with minimal variance signifies meticulous design and engineering of the spray mechanism, resulting in a nearly consistent volume of liquid dispensed during each spray. Such a consistent volume is of paramount importance because it plays a dual role in ensuring accurate dosing and forming an effective film over the target area. ^46^

The spray pattern, denoting the spray’s geometric shape in the cross section, is a defining attribute that characterizes the behavior of the delivered substance upon actuation. ^47^ The favorable spray pattern, indicative of the optimized solution’s ability to achieve a desirable and uniform cross-sectional shape, further contributes to the formulation’s potential for wound treatment.

Spray angles are essential to prevent over-spraying of the film-forming solution onto the skin. It also provides adequate coverage for product application. Gohel *et al*. stated that the suitable angle for spray determination was less than 85° for easy operation and covering the total surface area. ^48^

The amount of curcumin delivered with each actuation is of substantial importance, as it directly influences the uniformity of drug distribution and subsequently affects the therapeutic effectiveness of the formulation. The closely aligned quantity, accompanied by minimal variance, signifies meticulous engineering of the delivery mechanism and precise calibration.

The enhanced drug permeation observed with the film-forming spray formulation emphasizes its compatibility with the permeation characteristics of curcumin and its potential to enhance patient compliance owing to its convenient administration and transport attributes. This investigation underscores the superiority of the film-forming spray over liquid and semi-solid preparations, establishing its efficacy in terms of permeability.

## Conclusions

The formulation and evaluation of a curcumin film-forming spray for wound healing has shown promising results. Film-forming sprays offer several advantages over traditional topical formulations, including improved absorption, reduced skin irritation, and a consistent dose distribution. A molecular docking study augmented the anti-inflammatory properties of curcumin. The drug was successfully incorporated into a film-forming solution and sprayed with a propellant. The formulated solution exhibited favorable pH and viscosity and demonstrated prolonged permeation of curcumin with high *ex vivo* drug permeation. Notably, the formulation was found to be a non-irritant, ensuring its suitability for topical applications. This study presents a novel approach to effectively deliver curcumin locally for wound healing, while enhancing patient compliance using a film-forming spray. Further extensive research and clinical studies are warranted to explore the full potential of curcumin-based film formation in wounds and pain management.

## Authors contributions

AS, JC, and AJ conceptualized this study. AS and JC strategized the methods and AS supervised the work. JC and AN conducted the experiments and analyzed the data. AS, MM, PD, AD, and SH interpreted data. PD conceptualized the docking study. MM, AD, AN, and SH designed the figure and performed statistical analysis. All authors aided in drafting the manuscript. All the authors critically reviewed, edited, and approved the final manuscript.

## Data Availability

Figshare: Underlying data for ‘Fabrication and *in vitro* characterization of curcumin film-forming topical spray: An integrated approach for enhanced patient comfort and efficacy’, https://www.doi.org/10.6084/m9.figshare.24191067. ^27^ Figshare: ARRIVE checklist for ‘Fabrication and *in vitro* characterization of curcumin film-forming topical spray: An integrated approach for enhanced patient comfort and efficacy’, https://www.doi.org/10.6084/m9.figshare.24191067. ^27^ Data are available under the terms of the Creative Commons Attribution 4.0 International license (CC-BY 4.0)
