# Supplementary material for: Fabrication and in vitro characterization of curcumin film-forming topical spray: An integrated approach for enhanced patient comfort and efficacy
Source: F1000Res. 2024 Feb 22;13:138. [Version 1] doi: 10.12688/f1000research.142860.1 (PMC11109671; doi:10.12688/f1000research.142860.1)
Supplement: Peer review history

# Reviewer report: Reviewer response for version 1

**DOI:** 10.5256/f1000research.156459.r249577

**Published:** 2024-05-30

**Authors:** Ruchi Tiwari

**Affiliations:** 1 Pranveer Singh Institute of Technology, Kanpur, Uttar Pradesh, India

Strength

- The article “Fabrication and in vitro characterization of curcumin film-forming topical spray: An integrated approach for enhanced patient comfort and efficacy” is well within the journal’s scope.
- The paper introduces a novel method for delivering curcumin utilizing a film-forming spray, offering a convenient and precise means of administration.
- The study extensively characterizes the developed formulation, including its pH, viscosity, film formation time, drug content, and permeation properties, providing a thorough understanding of its characteristics.
- The study demonstrates superior drug permeation compared to traditional cream formulations and pure drug solutions, indicating the effectiveness of the developed film-forming spray in facilitating drug delivery.

Opportunity

- Avoid typographical errors such as and 2 times in the abstract.
- Inconsistency in the usage of time units; it alternates between “h” and “hr” and “sec” and “s.” Kindly maintain uniformity by choosing one format throughout the manuscript.
- Include information on the temperature and agitation speed of the shaking water bath during the solubility testing of curcumin in various solvents, as these factors can influence solubility, ensuring the experiment's reproducibility.
- Specify whether the stickiness evaluation and pH determination were conducted in triplicates, similar to the film formation time assessment, to provide information on the reproducibility and reliability of the experimental findings.
- Table 5 in the text says optimization results, whereas the skin irritation tests table has been given. Kindly rectify it.

Weaknesses:

- While the study demonstrates promising results in vitro, the absence of in vivo experimentation limits the ability to fully assess the efficacy and safety of the formulated spray in real physiological conditions. However, the authors acknowledge this limitation in the conclusion and suggest further extensive research and clinical studies to address this gap, indicating awareness of the need for further validation.

Overall, the topic is relevant. The manuscript is recommended for indexing with the changes to the above-mentioned comments.

Is the work clearly and accurately presented and does it cite the current literature?

Yes

If applicable, is the statistical analysis and its interpretation appropriate?

I cannot comment. A qualified statistician is required.

Are all the source data underlying the results available to ensure full reproducibility?

Yes

Is the study design appropriate and is the work technically sound?

Yes

Are the conclusions drawn adequately supported by the results?

Yes

Are sufficient details of methods and analysis provided to allow replication by others?

Yes

Reviewer Expertise:

Pharmaceutical sciences including all aspects of nano drug delivery systems.

I confirm that I have read this submission and believe that I have an appropriate level of expertise to confirm that it is of an acceptable scientific standard.

# Reviewer report: Reviewer response for version 1

**DOI:** 10.5256/f1000research.156459.r249576

**Published:** 2024-05-14

**Authors:** Tejal Mehta

**Affiliations:** 1 Department of Pharmaceutics, Institute of Pharmacy, Institute of Pharmacy, Nirma University, Ahmedabad, Gujrat, India

The work done systematically using experimental design. Results are justified with suitable cases. Following are the suggestions:

 1. Include units in Table 2

 2. Add the reason to choose particular grade of Eudragit in present study.

 3.  How the organic solvent residue is checked in final product?

Is the work clearly and accurately presented and does it cite the current literature?

Yes

If applicable, is the statistical analysis and its interpretation appropriate?

Yes

Are all the source data underlying the results available to ensure full reproducibility?

Yes

Is the study design appropriate and is the work technically sound?

Yes

Are the conclusions drawn adequately supported by the results?

Yes

Are sufficient details of methods and analysis provided to allow replication by others?

Yes

Reviewer Expertise:

Novel drug delivery systems and Pharmaceutical Technology

I confirm that I have read this submission and believe that I have an appropriate level of expertise to confirm that it is of an acceptable scientific standard.

# Reviewer report: Reviewer response for version 1

**DOI:** 10.5256/f1000research.156459.r259329

**Published:** 2024-04-18

**Authors:** Minakshi Nitin Nehete

**Affiliations:** 1 SNDT Women's University, Maharashtra, India

The article titled **"Fabrication and In vitro Characterization of Curcumin Film-Forming Topical Spray: An Integrated Approach for Enhanced Patient Comfort and Efficacy**" presents a well-written account with sufficient detail regarding methods and analysis. It serves as a valuable resource for researchers looking to undertake similar research endeavors.

 The **introduction** provides comprehensive details regarding the study, including background, rationale, and the selection of curcumin and film-forming spray. However, it fails to specify the types of wounds for which the developed curcumin film-forming spray is intended, such as acute, diabetic, etc.

 In the **methods **section, it is noted that each group of laboratory animals only consists of three rats. According to ICH guidelines, a treatment group should ideally contain six animals per group. Additionally, the name of the standard used in the skin irritation study is not mentioned.

 The article meticulously outlines the formulation and evaluation processes of the curcumin film-forming topical spray.

 The **results **section is detailed and informative.

 In the **discussion **section, formulation-related aspects are discussed thoroughly. However, the mechanism of action of the curcumin film-forming spray is not addressed in relation to wound healing activity, particularly concerning molecular docking study results.

 While the article does address some aspects of current literature, it falls short in fully addressing others. Furthermore, in the references section, while most references are from current literature, a few are not up-to-date.

Is the work clearly and accurately presented and does it cite the current literature?

Partly

If applicable, is the statistical analysis and its interpretation appropriate?

Yes

Are all the source data underlying the results available to ensure full reproducibility?

Yes

Is the study design appropriate and is the work technically sound?

Partly

Are the conclusions drawn adequately supported by the results?

Yes

Are sufficient details of methods and analysis provided to allow replication by others?

Yes

Reviewer Expertise:

phytochemical analysis, phytochemistry, pharmacology, molecular docking study

I confirm that I have read this submission and believe that I have an appropriate level of expertise to confirm that it is of an acceptable scientific standard.
